# Pathophysiological Role of Chymase-Activated Matrix Metalloproteinase-9

**DOI:** 10.3390/biomedicines10102499

**Published:** 2022-10-07

**Authors:** Shinji Takai, Denan Jin

**Affiliations:** Department of Innovative Medicine, Osaka Medical and Pharmaceutical University, Osaka 569-8686, Japan

**Keywords:** chymase, matrix metalloproteinase-9, inhibitor

## Abstract

Chymase present in mast cells can directly form matrix metalloproteinase (MMP)-9 from proMMP-9. Chymase-activated MMP-9 has been reportedly closely related to the pathogenesis of various diseases, and inflammation-related diseases in particular. Upregulated chymase and MMP-9 have been observed in tissues from patients and animal models of aortic aneurysm, inflammatory gastrointestinal and hepatic diseases, acute pancreatic failure, atopic dermatitis and rheumatoid arthritis. Chymase at these regions is only derived from mast cells, while MMP-9 is derived from macrophages and neutrophils in addition to mast cells. Chymase inhibitors attenuate MMP-9 formation from pro-MMP-9, and ameliorate the development and progression of these disorders, along with reduction in inflammatory cell numbers. MMP-9 activated by chymase might also be involved in angiogenesis in the tumor environment. Development of angiogenesis around several cancers is closely related to the expression of chymase and MMP-9, and postoperative survival curves have revealed that patients with a higher number of chymase positive cells have lower survival rates. In this review, we wanted to clarify the role of chymase-activated MMP-9, which might become an important therapeutic target for various inflammatory disorders.

## 1. Introduction

**Figure 1:**
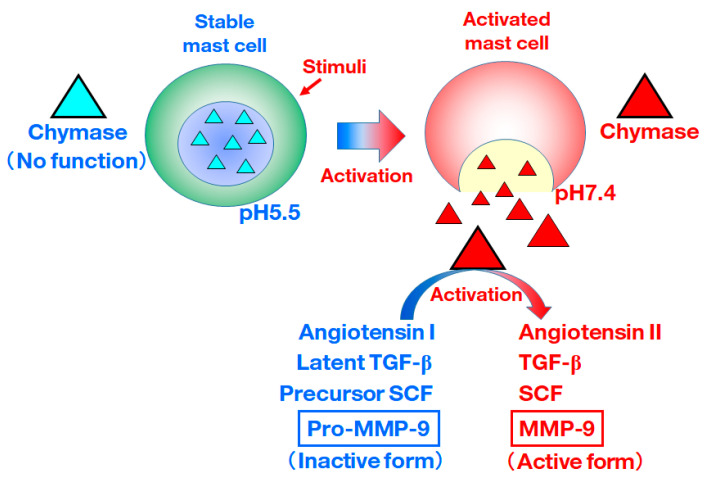
Chymase is stored in the secretory granules of inactive mast cells. The pH within granules is maintained at 5.5, at which the chymase has no enzymatic activity. Chymase exerts its enzymatic functions, such as activation of angiotensin II, TGF-β, SCF and MMP-9 from their respective precursors, upon release from mast cell granules, following activation by various stimuli.

Chymase (EC 3.4.21.39) is a chymotrypsin-like serine protease that is present in secretory granules under normal conditions. The pH within secretory granules is regulated at 5.5 [1]. Hence, chymase has no enzymatic activity in the granules, because the optimal pH of chymase is between 7 and 9 [2]. Stimulation of mast cells, such as by inflammation, oxidative stress, and mechanical stress, causes the release of chymase from the granules of mast cells, leading to its various enzymatic activities (Figure 1). However, due to the activity of endogenous chymase inhibitors, such as α-antitrypsin and α-antichymotrypsin, found in blood, the enzymatic function of chymase disappears immediately. Additionally, although mast cells are known to be expressed and distributed within most tissues throughout the body, chymase has no enzymatic activity in normal tissues. Therefore, chymase can only exert its enzymatic function in inflamed and injured tissues where mast cells are activated.

The enzymatic function of chymase is similar to that of chymotrypsin, which cleaves the C-terminal of aromatic amino acids such as phenylalanine (Phe), tyrosine (Tyr) and tryptophan (Trp). In a previous animal study, dog mastocytoma cells produced pro-matrix metalloproteinase (MMP)-9, which was converted to MMP-9 by cleavage of the Phe88–Gln89 and Phe91–Glu92 bonds by purified dog chymase [3]. In an extract from human aneurysmal aortas as well, purified human chymase was found to cleave the Phe91–Glu92 bond of pro-MMP-9 to form MMP-9 [4]. The cleavage sites of pro-MMP-9 by chymase might, however, vary between species.

In another study in chymase-deficient mice, the conversion of pro-MMP-9 to MMP-9 was abolished in various tissues, with this phenomenon indicating the significance of chymase in the activation of pro-MMP-9 to MMP-9 [5]. MMP-9 is a zinc-dependent enzyme with proteolytic activity against extracellular matrixes that also serves as a key enzyme in tissue remodeling. As the enzymatic functions of chymase other than activation of MMP-9 from pro-MMP-9, activation of angiotensin II, transforming growth factor (TGF)-β, stem cell factor (SCF) and MMP-9 from their respective precursors. TGF-β plays a crucial role in the development and progression of tissue fibrosis. Angiotensin II plays an important role in regulating blood pressure via its functions, such as in vascular contraction and fluid retention, but it can also promote the proliferation of fibroblasts via the induction of gene expression of TGF-β [6]. The activation of SCF from the inactive membrane-bound form of SCF is also important as an enzymatic function of chymase [6]. SCF, termed “mast cell growth factor”, is an essential cytokine and is necessary for differentiation and proliferation via stimuli of its receptor c-KIT at the surface of immature and mature mast cells, respectively [7]. Chymase-dependent SCF activation may be involved in the induction of mast cell proliferation. This review mainly focuses on the pathophysiological role of chymase-activated MMP-9 in various diseases.

## 2. Relationship between MMP-9 and Various Diseases

**Figure 2:**
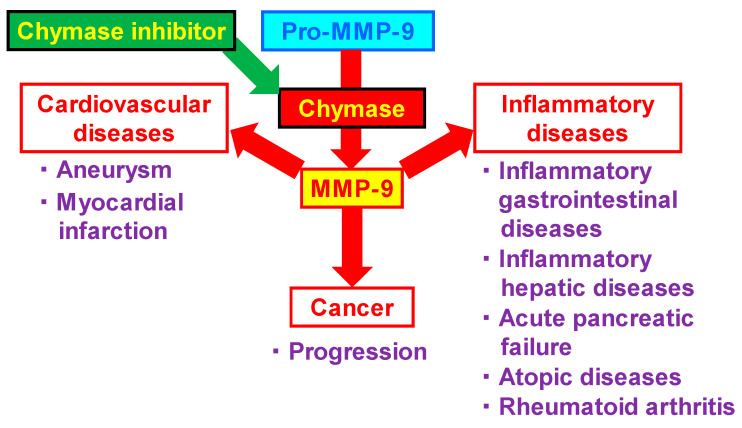
Relationship between MMP-9 and diseases. MMP-9 promotes the cardiovascular diseases such as aneurysm and cardiac remodeling after myocardial infarction. MMP-9 is involved in inflammatory diseases via the accumulation of inflammatory cells. MMP-9 may also play an important role in the cancer progression via the induction of angiogenesis at the surround of tumor. Chymase inhibitor is a useful strategy to prevent chymase-activated MMP-9.

MMP-9 has been implicated in a number of diseases, including cardiovascular diseases, inflammatory disorders, and cancers. MMP-9 promotes the infiltration of inflammatory cells into vascular tissues by cleaving intercellular adhesion molecules, such as fibronectin and vitronectin. Their MMP-9-mediated actions might be involved in the pathogenesis of aortic and intracranial aneurysms and their rupture. In terms of the association between cardiac disease and MMP-9, MMP-9 might be profoundly involved in cardiac remodeling immediately after myocardial infarction. There are many associations between MMP-9 and inflammatory diseases, including inflammatory gastrointestinal, inflammatory hepatic, atopic diseases, acute pancreatic failure, and rheumatoid arthritis. Macrophages and neutrophils are well known as MMP-9 expressing cells, although it is also expressed on mast cells. Therefore, MMP-9 expression is increased in lesions with accumulation of these inflammatory cells, and suppression of the MMP-9-mediated vicious cycle is considered to be an important therapeutic target in inflammatory diseases. In cancers, MMP-9 might have a greater impact on the surround of tumor than on the cancer cells themselves, resulting in a deeper involvement in cancer growth. Chymase inhibitor is a useful strategy to prevent chymase-activated MMP-9 and is expected to show efficacy against a variety of diseases (Figure 2).

## 3. Cardiovascular Diseases

### 3.1. Aneurysm

Chymase has been well known to activate angiotensin I to angiotensin II, and many studies of angiotensin II produced by chymase in cardiovascular diseases has been reported. On the other hand, the role of chymase-activated MMP-9 is deeply involved in inflammatory cell accumulation, which may be particularly relevant to inflammation-associated cardiovascular diseases such as aneurysm. Aortic aneurysms are generally characterized by an inflammatory response in and widespread destruction of the aortic media. The pathophysiology of aortic aneurysm represents an imbalance between the production and degradation of structural extracellular matrix proteins. In the normal aorta, elastic fibers maintain the vascular wall structure, while augmentation of MMP-9 expression leads to the elastic fiber disruption seen in human aortic aneurysms [8]. In a previous study, progression of aortic aneurysms induced by elastase was significantly attenuated in MMP-9-deficient mice [9]. Therefore, MMP-9 is thought to play a critical role in the progression of aortic aneurysms.

Augmentation of chymase activity has been observed in human aortic aneurysms [10]. MMP-9 activity was also time-dependently increased in an extract of human aortic aneurysms [4], suggesting the existence of MMP-9-activating enzymes in extracts from human aortic aneurysms. Further, MMP-9 activity in the extract was decreased by 50% by a chymase inhibitor, although the addition of purified human chymase to the extract increased MMP-9 activity 1.7-fold [4]. The cleavage site of pro-MMP-9 to MMP-9 by dog chymase is the Phe^91^–Glu^92^ bond [4]. On the other hand, human chymase cleaves the Phe^88^–Gln^89^ and Phe^91^–Glu^92^ bonds of Pro-MMP-9 [3]. In a dog elastase-induced aortic aneurysm model, both MMP-9 and chymase activities were significantly increased, and chymase inhibition significantly attenuated expansion of the aortic aneurysm, along with a reduction in chymase and MMP-9 activity [11]. In another study, although the cleavage site of pro-MMP-9 was unclear, the elastase-induced aortic aneurysm was significantly attenuated in mouse chymase-deficient mice [5]. Significant increases in chymase and MMP-9 activities were also observed in a mouse aortic aneurysm model that was developed by continuous administration of angiotensin II to apolipoprotein E-deficient mice, and expansion of the aortic aneurysm was also prevented by chymase inhibitors [12,13]. In a mouse model of intracranial aneurysm rupture, a significant increase in the rupture rate was observed by treatment with a mast cell activator that induces several factors, including chymase release from mast cells, while, on the other hand, a preventive effect on aneurysmal rupture was observed by administration of a mast cell stabilizer that inhibits chymase release [14]. Thus, MMP-9 activation by chymase might be involved in the progression and rupture of aneurysms via degradation of the matrix of vascular structures.

Rupture of an intracranial aneurysm results in aneurysmal subarachnoid hemorrhage. The 30-day mortality rate after aneurysmal subarachnoid hemorrhage reaches 45% [15]. A recent paper demonstrates that mast cell induces the intracranial aneurysm rupture [14]. In a mouse model with intracranial aneurysms, a mast cell stabilizer markedly decreased the rupture rate of aneurysms, but a mast cell activator significantly increased it. Moreover, the rupture rate of aneurysms was significantly reduced in mast cell-deficient mice [14]. On the other hand, augmentation of MMP-9 has been observed in the lesion of intracranial aneurysm rupture in patients and a rat model [16,17]. Chymase-activated MMP-9 may also contribute to the intracranial aneurysm rupture.

### 3.2. Myocardial Infarction

Atherosclerosis is a major cause of coronary artery diseases. The process starts with the accumulation of lipids, leading to promotion of the development and progression of plaques. MMPs play a key role in the pathogenesis of atherosclerosis through low-density lipoprotein recruitment, and migration of vascular smooth muscle cells and inflammatory cells into endothelium via extracellular matrix degradation [18]. Plaque rupture or erosion also induces myocardial infarction, and ruptured plaques are characterized by a large lipid-rich core, a thin fibrous cap and many inflammatory cells, such as macrophages, which express MMP-9 [19]. A large number of mast cells and MMP-9-positive macrophages have been demonstrated in the coronary artery lesions of unstable angina patients [20]. Plasma MMP-9 levels were also seen to be sharply increased just after acute myocardial infarction in patients [21]. Thus, MMP-9 might be associated with the plaque rupture of coronary arteries, and its activation by chymase might also play an important role in the pathogenesis of myocardial infarction.

In animal studies as well, both MMP-9 and chymase activities in the infarcted cardiac tissues were significantly increased in the acute phase just after myocardial infarction in hamsters [22,23]. In contrast, cardiac dysfunction after myocardial infarction was significantly attenuated by an MMP-9 inhibitor or a chymase inhibitor [22,23]. Further, a reduction in macrophage infiltration in infarcted cardiac lesions after myocardial infarction was observed in MMP-9-deficient mice compared with wild-type mice [24]. In a swine acute myocardial ischemia/reperfusion model, the areas of necrosis and fibrosis in the ischemic risk areas were significantly reduced along with attenuation of MMP-9 activity, in addition to inhibition of chymase activity, by a chymase inhibitor [25]. In another study using chymase-deficient mice, cardiac MMP-9 activity was markedly reduced compared to that in wild-type mice after myocardial infarction [26]. Moreover, cardiac dysfunction and survival rates were significantly improved in chymase-deficient mice [27]. These findings strongly suggest the importance of MMP-9 attenuation by chymase inhibition in the prevention of cardiac remodeling at acute phase after myocardial infarction. 

On the other hand, at chronic phase after myocardial infarction, in a rat myocardial infarction model, a chymase inhibitor significantly reduced mRNA levels of TGF-β, collagen I, and collagen III, in addition to chymase activity, in the left ventricle, thus improving cardiac dysfunction and fibrosis [28]. Chymase may be involved in the pathogenesis of cardiac fibrosis via the formation of angiotensin II and TGF-β. In autopsied patients with hypertensive heart diseases, chymase-positive mast cell numbers in the heart were significantly higher than in those without heart diseases, and a significant positive correlation between chymase-positive mast cell numbers and collagen fibers was found [29]. In canine heart failure induced by rapid right ventricular pacing, the number of chymase-positive mast cells increased in the left ventricle compared with the normal group; however, a chymase inhibitor significantly decreased chymase-positive mast cell density, cardiac angiotensin II concentration, mRNA levels of TGF-β, collagen I, and collagen III, and cardiac fibrosis [30]. Increases in chymase-dependent angiotensin II and TGF-β formation may be involved in the development of cardiac fibrosis observed at chronic phase after myocardial infarction. 

## 4. Inflammatory Diseases

### 4.1. Inflammatory Gastrointestinal Diseases

*Helicobacter pylori* plays an important role in the pathogenesis of chronic gastritis, gastric ulcers and gastric adenocarcinoma, although most individuals infected with *Helicobacter pylori* develop only superficial gastritis. In gastric epithelial cells, MMP-9 gene expression is reportedly significantly increased after *Helicobacter pylori* infection [31], as seen by the fact that the MMP-9 gene expression by epithelial cells from patients infected with *Helicobacter pylori* was significantly augmented, compared with that by cells from non-infected patients [31]. Additionally, augmentation of the chymase-positive mast cell number was observed in regions in human stomachs with *Helicobacter pylori*-associated gastritis [32]. Although a direct interaction between MMP-9 and chymase in gastritis has not been reported, the role of MMP-9 activation by chymase in the development of gastritis should be studied in the future.

Nonsteroidal anti-inflammatory drugs (NSAIDs) such as indomethacin have been widely used as anti-inflammatory therapy, but are known to produce gastric ulcers. Malvidin is an anthocyanidin present in grapes, wine, and black rice, that has been reported to contribute to the prevention of cardiovascular diseases and diabetes, along with its anti-inflammatory and antioxidant activities. Malvidin has also been suggested to prevent indomethacin-induced gastric ulcers in mice via MMP-9 reduction, in addition to its anti-oxidative effects [33]. NSAIDs induce not only gastric ulcer formation, but also intestinal injury. Since it is now possible to examine the small intestine due to the development of capsule endoscopy and double-balloon endoscopy, NSAID-induced injury in the small intestine has been clarified. In an NSAID diclofenac sodium-induced intestinal injury model in mice, mast cell number was significantly increased in the area of intestinal lesions [34]. Further, both chymase and MMP-9 activities were significantly augmented in the lesions in a rat indomethacin-induced small intestinal injury model, and a significant reduction in the injured area and significant attenuation of chymase and MMP-9 activities were observed following treatment with a chymase inhibitor [35]. In this model, the Pro-MMP-9 level was reduced in the extract from the injured lesion, albeit with an increase in MMP-9 levels [35]. On the other hand, a significant increase in Pro-MMP-9 levels and a significant decrease in MMP-9 levels were shown following treatment with a chymase inhibitor [35]. These findings suggest that chymase might play an important role in the activation of MMP-9 from Pro-MMP-9 in indomethacin-induced small intestinal injury.

Ulcerative colitis is an inflammatory bowel disease (IBD) affecting the distal colon, in which the recruitment of inflammatory cells induces degradation of extracellular matrix. MMPs are important enzymes in the degradation of extracellular matrix proteins, and are involved in the pathogenesis of IBD via extracellular matrix degradation [36]. MMP-2 and MMP-9 levels were consistently up-regulated during active flare-ups of IBD in patients and an animal colitis model [37,38]. In previous studies, oral administration of dextran sodium sulfate (DSS) was used to induce colitis in an animal model, which resembled the lesions observed in patients with ulcerative colitis. DSS-induced colitis was found to be significantly attenuated in MMP-9-deficient mice, but not in MMP-2-deficient mice [39,40]. Thus, MMP-9 rather than MMP-2 might play an important role in the pathogenesis of DSS-induced colitis via degradation of extracellular matrix proteins in ulcerative colitis. In a DSS-induced colitis mouse model, not only MMP-9 activity but also chymase activity was significantly increased in the colitis lesions [41]. Further, a chymase inhibitor reduced both chymase and MMP-9 activities and attenuated body weight loss, amount of fecal blood and shortening of the colon, in addition to lower histological damage scores [41]. Moreover, the increase in MMP-9 levels after incubation of an extract of colitis lesions was completely prevented by treatment with a chymase inhibitor [41]. This suggests that chymase-dependent MMP-9 activation might play a crucial role in the development of DSS-induced colitis.

### 4.2. Inflammatory Hepatic Diseases

Acute liver failure is induced by viral infections, excessive alcohol intake, and certain drugs. Although it is associated with a high mortality, there are no effective therapeutic strategies for acute liver failure. Activation of T cells and macrophages are observed in the initiation of acute liver failure, which induce the release of proinflammatory cytokines, such as tumor necrosis factor (TNF)-α. In a previous animal study, the combined administration of lipopolysaccharide (LPS) and D-galactosamine was used to induce acute liver failure in an animal model. The amino sugar D-galactosamine is selectively metabolized by hepatocytes and induces transcriptional inhibition in the liver [42]. LPS is the primary component of the endotoxins of Gram-negative bacteria and is potentiated by D-galactosamine, resulting in acute liver failure [43]. In a mouse acute liver failure model, an MMP inhibitor reduced alanine aminotransferase (ALT) and attenuated macrophage and neutrophil accumulation [44]. Significant attenuation of acute liver failure symptoms after the combined administration of LPS and D-galactosamine was also observed in MMP-9-deficient mice [45]. In acute liver failure induced by LPS and D-galactosamine in hamsters, augmentation of chymase and MMP-9 activities were observed, although they were dose-dependently attenuated by treatment with a chymase inhibitor [46]. In that study, significant increases in ALT, aspartate aminotransferase (AST) and the hepatic necrotic area were also abolished by chymase inhibition [46]. This indicates that the development of acute liver failure might be a result of MMP-9 activation by chymase, and that chymase inhibition might be a useful strategy for attenuation of acute liver failure symptoms.

Non-alcoholic fatty liver disease (NAFLD) is characterized by excessive fat accumulation in the liver that is unrelated to alcohol consumption and viral infection. Approximately 10% of NAFLD patients develop into non-alcoholic steatohepatitis (NASH), which is characterized by inflammation and fibrosis and progress to cirrhosis and hepatocellular carcinoma [47,48]. The progression of NAFLD and the development of NASH are associated with the metabolic syndrome resulting from obesity, diabetes, hypertension and hyperlipidemia [49,50,51]. NAFLD presents as simple hepatic steatosis, and NASH is characterized by lobular inflammation, severe steatosis and liver fibrosis [52]. In a rat high-fat and high-cholesterol (HFC) diet-induced NASH model, significant increases in chymase and MMP-9 activity were observed in livers with NASH, and the development of steatosis, inflammatory cell accumulation, and fibrosis in the liver was significantly prevented by administration of a chymase inhibitor before the HFC diet [53]. Furthermore, a significant improvement of NASH was also observed even when the chymase inhibitor therapy was initiated after the complete development of NASH [53]. Chymase is able to form angiotensin II and TGF-β from angiotensin I and the precursor of TGF-β, respectively, along with MMP-9 activation. Both angiotensin II and TGF-β are closely associated with the development and progression of tissue fibrosis in NASH. Therefore, attenuation of angiotensin II and TGF-β, in addition to the reduction in MMP-9 by chymase inhibition, might contribute to preventing and improving NASH.

### 4.3. Acute Pancreatic Failure

Acute pancreatitis is an inflammatory disease triggered by autolysis due to the activation of pancreatic enzymes. Although the mortality of acute pancreatitis has decreased to less than 5%, as described in international guidelines, the mortality of severe acute pancreatitis is still 10–20% [54,55]. Most guidelines for acute pancreatitis only mention supportive therapies, such as fluid management and nutritional intervention, and there is no reliable therapeutic agent that can be recommended at present [54,55]. In a rat taurodeoxycholate-induced acute pancreatitis model, acute pancreatitis was observed along with mast cell activation and an increase in TNF-α level in the pancreas, although these were prevented by pretreatment with a mast cell stabilizer [56]. In a rat cerulein-induced acute pancreatitis model, mast cell numbers significantly increased in the region of pancreatitis, although a significant reduction in neutrophil infiltration was observed following treatment with a mast cell stabilizer [57]. These results suggest the significance of mast cells in initiating acute pancreatitis. MMP-9 activity was also significantly increased in the pancreas in acute pancreatitis animal models, and the severity of pancreatitis was significantly attenuated by treatment with an MMP inhibitor and in MMP-9-deficient mice [58].

In an L-arginine-induced hamster pancreatitis model, neutrophil infiltration of the pancreas was observed along with significant increases in serum lipase and TNF-α from 1 h after L-arginine administration [59]. In that model, both chymase and MMP-9 activities in the pancreas were significantly increased, although they were significantly attenuated by a chymase inhibitor. Furthermore, the survival rate was significantly higher in the chymase-treated group than the placebo-treated group in the L-arginine-induced hamster pancreatitis model. Thus, chymase inhibition might become a useful strategy for attenuating the symptoms of, and reducing death associated with, acute pancreatitis. 

### 4.4. Atopic Diseases

Atopic dermatitis is a chronic inflammatory skin disease and induces the development of rhinitis, conjunctivitis and asthma. The pathogenesis of atopic dermatitis is thought to involve abnormality of the immune system, such as IgE-mediated sensitization [60]. The number of chymase-positive mast cells in skin is reportedly increased in patients with atopic dermatitis [60]. Inflammatory cells such as neutrophils and eosinophils accumulate at the site after the injection of purified chymase into human or rat skin [61,62]. Atopic dermatitis in NC/Nga mice has been widely used as an atopic model. In NC/Nga mice, a chymase inhibitor improved the atopic dermatitis by minimizing the accumulation of inflammatory cells such as eosinophils, neutrophils, T lymphocytes and mast cells [63,64]. A phase II clinical trial of a chymase inhibitor was conducted in patients with atopic dermatitis in a randomized, placebo-controlled, double-blind study [65]. Mean improvement in eczema area and severity index (EASI) was 47% and 38% in the chymase-inhibitor- and placebo-treated patients, respectively, and the difference was statistically significant; *p* = 0.006 [65]. A subset of atopy probable patients (plasma IgE > 100 IU/mL) showed enhanced response, with mean improvement in EASI of 52% and 28% in the chymase inhibitor- and placebo-treated patients, respectively (*p* = 0.001) [65]. On the other hand, the side effects with the chymase inhibitor were similar to the placebo, and no significant difference [65].

Chymase-activated MMP-9 has been shown to be involved in ocular surface damage via degradation of extracellular matrix in corneal cells [66,67]. The development of vernal keratoconjunctivitis is also deeply associated with atopic dermatitis and asthma. It has been reported that the more severe the disease, the higher the chymase activity in tears from patients with vernal keratoconjunctivitis, and the more significant the correlation between the degree of chymase activity and the severity of vernal keratoconjunctivitis [66]. In patients with vernal keratoconjunctivitis, tear pro-MMP-9 level was significantly higher than normal control subjects, and MMP-9 activity correlated significantly with corneal involvement and giant papillae formation [67]. In a guinea pig model of allergic conjunctivitis, chymase activity was increased in ophthalmic lavage fluid, and the increased histamine in the ophthalmic lavage fluid was inhibited by a chymase inhibitor [68]. Chymase might thus be a potential pharmacotherapeutic target for atopic diseases, including allergic conjunctivitis.

### 4.5. Rheumatoid Arthritis

Rheumatoid arthritis is an autoimmune disease that presents with joint swelling and pain due to synovial inflammation. The main component of the synovial matrix is hyaluronic acid, which plays an important role as a lubricator in joints. MMP-9 is upregulated in the synovial fluid in patients with rheumatoid arthritis [69]. In the joint fluid of rheumatoid arthritis patients, a significant correlation between MMP-9 and polymorphonuclearleukocyte elastase levels was observed, and MMP-9 level was also correlated with the C-propeptide of type II collagen level, which reflects the synthesis of type II collagen and is up-regulated angiogenesis [70]. In a rat arthritis model, hyaluronic acid concentration was decreased, and MMP-9 activity was increased in synovial fluid [71]. In that model, chymase activity was significantly increased in the synovial fluid, and a significant positive correlation between chymase and MMP-9 activities was observed [71]. Furthermore, hyaluronic acid concentration correlated negatively with chymase and MMP-9 activities [71]. A mast cell stabilizer significantly attenuated the joint destruction in a mouse arthritis model [72]. The MMP-9 level was significantly increased in synovial tissues after the chymase injection [73]. In patients with rheumatoid arthritis, a significant increase in chymase and a significant decrease in hyaluronic acid concentration have also been observed [71]. The decrease in hyaluronic acid concentration in synovial fluid might be associated with the increase in chymase. However, it is unclear whether chymase inhibitors are effective for experimental arthritis models and patients with rheumatoid arthritis.

## 5. Cancer

The tumor microenvironment (TME) is closely related to immune cells, including mast cells and angiogenesis. Enhanced angiogenesis by vascular endothelial growth factor (VEGF) plays an important role in the optimum TME for cancer progression. In mast cell-deficient mice, angiogenesis was lower than that in normal wild mice, and metastasis to the lung was also attenuated after transplantation of melanoma [74]. In azoxymethan and DSS-induced colon cancer, mast cell-deficient mice exhibited significant less tumor formation than normal mice [75]. Both the number of microvessels and chymase-positive mast cells were increased in pulmonary cancers, and there is a significant positive correlation between their numbers in the TME collected from such patients [76]. A significant positive correlation between chymase-positive mast cells and microvessel numbers was reported in patients with pancreatic cancer [77]. A significant increase in microvessel and chymase-positive mast cell numbers were also observed in patients with gastric cancer, and postoperative survival curves revealed that patients with higher numbers of chymase-positive mast cells had the lowest survival rates [78]. 

**Figure 3:**
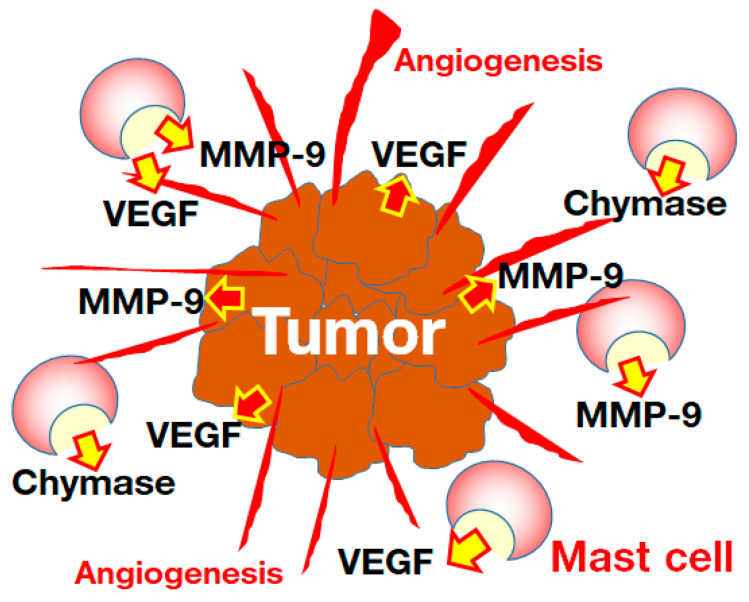
Tumors secrete VEGF and MMP-9, both of which contribute to angiogenesis in the TME; mast cells also release VEGF and MMP-9, in addition to chymase, after their activation.

Chymase is able to convert angiotensin I to angiotensin II, which has a potent angiogenic action via the induction of VEGF mRNA level [79]. In hamsters, treatment with a chymase gene or its protein via a sponge embedded in the back strongly facilitated angiogenesis [80]. In contrast, a chymase inhibitor was able to prevent angiogenesis via the attenuation of up-regulated VEGF mRNA level induced by basic fibroblast growth factor in hamsters [80]. Although tumors are known to secrete VEGF and MMP-9, both of which contribute to angiogenesis in the TME, mast cells also release VEGF and MMP-9 in addition to chymase, after their activation [81]. Chymase also converts pro-MMP-9 to MMP-9, which is also an angiogenic factor in the TME [82]. In a previous study, the increase in microvessel numbers in the TME was significantly attenuated in MMP-9 knockout mice [83]. Chymase might, thus, contribute to the progression of tumor growth via the facilitation of angiogenesis in the TME (Figure 3). In the future, it is necessary to confirm whether chymase inhibition might be effective therapy against tumors via improvement of the TME. 

## 6. Conclusions

Chymase-activated MMP-9 has been shown to be involved in the development and progression of cardiovascular and inflammatory diseases and cancer in patients and experimental models. A chymase inhibitor for prevention of heart failure after myocardial infarction and diabetic renal failure has been tested in clinical phase II studies, and while it was shown to be very safe, no clear efficacy has been demonstrated [84,85]. One reason for the lack of efficacy might have been the large number of patients treated with angiotensin-converting enzyme (ACE) inhibitors or angiotensin II receptor blockers (ARBs). Angiotensin II plays an important role in the pathogenesis of cardio-renal failure, and the additional benefits of administering a chymase inhibitor to patients receiving ACE inhibitors/ARBs might be limited [84,85]. On the other hand, a chymase inhibitor has shown efficacy for atopic dermatitis in a phase II clinical study [65]. Chymase inhibitors might, thus, be effective against inflammatory disorders in which MMP-9 is highly involved in the pathogenesis.

## Data Availability

Not applicable.
